# Impact of Preservation Method and 16S rRNA Hypervariable Region on Gut Microbiota Profiling

**DOI:** 10.1128/mSystems.00271-18

**Published:** 2019-02-26

**Authors:** Zigui Chen, Pak Chun Hui, Mamie Hui, Yun Kit Yeoh, Po Yee Wong, Martin C. W. Chan, Martin C. S. Wong, Siew C. Ng, Francis K. L. Chan, Paul K. S. Chan

**Affiliations:** a Centre for Gut Microbiota Research, Faculty of Medicine, The Chinese University of Hong Kong, Hong Kong SAR, China; b Department of Microbiology, Faculty of Medicine, The Chinese University of Hong Kong, Hong Kong SAR, China; c Jockey Club School of Public Health and Primary Care, Faculty of Medicine, The Chinese University of Hong Kong, Hong Kong SAR, China; d Department of Medicine and Therapeutics, Faculty of Medicine, The Chinese University of Hong Kong, Hong Kong SAR, China; e LKS Institute of Health Science, State Key Laboratory of Digestive Disease, Faculty of Medicine, The Chinese University of Hong Kong, Hong Kong SAR, China; Northern Arizona University

**Keywords:** 16S rRNA gene, amplicon sequencing, bacterial culture, gut microbiota, preservative

## Abstract

Proper preservation of stool samples to minimize microbial community shifts and inactivate infectious agents is important for self-collected specimens requiring shipment to laboratories when cold chain transport is not feasible. In this study, we evaluated the performance of six preservation solutions (Norgen, OMNI, RNAlater, CURNA, HEMA, and Shield) for these aspects. Following storage of human stool samples with these preservatives at room temperature for 7 days, three hypervariable regions of the bacterial 16S rRNA gene (V1-V2, V3-V4, and V4) were amplicon sequenced. We found that samples collected in two preservatives, Norgen and OMNI, showed the least shift in community composition relative to −80°C standards compared with other storage conditions, and both efficiently inhibited the growth of aerobic and anaerobic bacteria. RNAlater did not prevent bacterial activity and exhibited relatively larger community shift. Although the effect of preservation solution was small compared to intersubject variation, notable changes in microbiota composition were observed, which could create biases in downstream data analysis. When community profiles inferred from different 16S rRNA gene hypervariable regions were compared, we found differential sensitivity of primer sets in identifying overall microbial community and certain bacterial taxa. For example, reads generated by the V4 primer pair showed a higher alpha diversity of the gut microbial community. The degenerate 27f-YM primer failed to detect the majority of *Bifidobacteriales*. Our data indicate that choice of preservation solution and 16S rRNA gene primer pair are critical determinants affecting gut microbiota profiling.

**IMPORTANCE** Large-scale human microbiota studies require specimens collected from multiple sites and/or time points to maximize detection of the small effects in microbe-host interactions. However, batch biases caused by experimental protocols, such as sample collection, massively parallel sequencing, and bioinformatics analyses, remain critical and should be minimized. This work evaluated the effects of preservation solutions and bacterial 16S rRNA gene primer pairs in revealing human gut microbiota composition. Since notable changes in detecting bacterial composition and abundance were observed among choice of preservatives and primer pairs, a consistent methodology is essential in minimizing their effects to facilitate comparisons between data sets.

## Abstract (precis)

Large-scale human microbiota studies require specimens collected from multiple sites and/or time points to maximize detection of the small effects in microbe-host interactions. However, batch biases caused by experimental protocols, such as sample collection, massively parallel sequencing, and bioinformatics analyses, remain critical and should be minimized. This work evaluated the effects of preservation solutions and bacterial 16S rRNA gene primer pairs in revealing human gut microbiota composition. Since notable changes in detecting bacterial composition and abundance were observed among choice of preservatives and primer pairs, a consistent methodology is essential in minimizing their effects to facilitate comparisons between data sets.

## INTRODUCTION

Humans harbor an enormous variety of bacteria, archaea, fungi, and viruses that form a community playing important roles in our metabolism and immune system (1, 2). Surveys of microbiomes inside the gastrointestinal tract, where most host-associated microbes reside, indicate that the disruption of microorganisms and their abundances could be linked to a number of diseases, such as carcinogenesis (3), cardiovascular disease (4), diabetes (5), hypertension (6), inflammatory bowel disease (7), and obesity (8). The study of gut microbiome has been empowered in a culture-independent manner through high-throughput sequencing and bioinformatics analyses (9–11). Because composition of gut microbiota differs greatly between individuals, microbiome studies have expanded into larger populations at multiple sites and/or time points to maximize detection of the small effects of microbe-host interactions. However, biases caused by methods of sample collection and choice of 16S rRNA gene hypervariable region remain critical and should be minimized.

Immediately freezing stool samples at −20°C or below is considered the “gold standard” to prevent shifting of microbial community composition (12). However, this approach is not feasible for sample collection in remote areas without reliable cold chain transport, or in studies where subjects are requested to send self-collected household samples to laboratories at ambient temperature. The effects of several storage conditions in revealing microbiota composition have been compared to immediate freezing at −80°C. For example, stool samples stored at room temperature for at most 1 day, or at 4°C and −20°C for up to 2 weeks, had little effect on microbiota composition shift (13–15). When samples are exposed to room temperature or higher other than ultralow freezing, however, use of preservation methods becomes essential and critical. Ethanol in concentrations of 95% or higher preserved bacterial DNA for long-term storage well (15), but another study has reported low DNA yield as a potential drawback of using ethanol (16). In addition, self-collected household samples stabilized with ethanol (70% and above) might require special shipping, depending upon local transportation regulations. RNAlater, commonly used as a general preservative for RNA samples, has been shown to have decreased DNA purity and to perform relatively poorly in maintaining microbiota composition derived by 16S rRNA gene amplicon sequencing (16, 17). Buffers containing EDTA can inhibit the growth of certain bacteria (18), but they significantly influenced gut microbiota profiles, such as an increased abundance of *Bacteroides* and *Proteobacteria* and a reduction of *Firmicutes* and *Actinobacteria* (13). OMNIgene.Gut, a commercially available approach to preserve stool samples at ambient temperature, has recently been reported to generate community profiles that diverge the least from −80°C standards (13, 19). However, its effectiveness relative to other approaches needs to be independently assessed over longer time scales. Moreover, these prior studies have generally not evaluated the bactericidal ability of preservatives in rendering microbiota samples noninfectious.

Since microbiome research is quickly shifting toward determination of small effect size variations relevant to host health, understanding the effects of preservative solutions is crucial to minimize bias. As part of the protocol development for a multiple-site, large-scale longitudinal microbiota study, we evaluated the effects of six preservative solutions (Norgen, OMNI, RNAlater, CURNA, HEMA, and Shield) in retaining gut microbiota composition compared to immediate freezing at −80°C. Stool samples from five healthy individuals were collected and subjected to storage in different preservation solutions for 7 days, a time period typically sufficient for delivery to the laboratory by post. Three hypervariable regions of the bacterial 16S rRNA gene (V1-V2, V3-V4, and V4) were amplicon sequenced. The bactericidal ability of these preservatives was assessed by standard aerobic and anaerobic cultures.

## RESULTS

### Study subjects.

**FIG 1:**
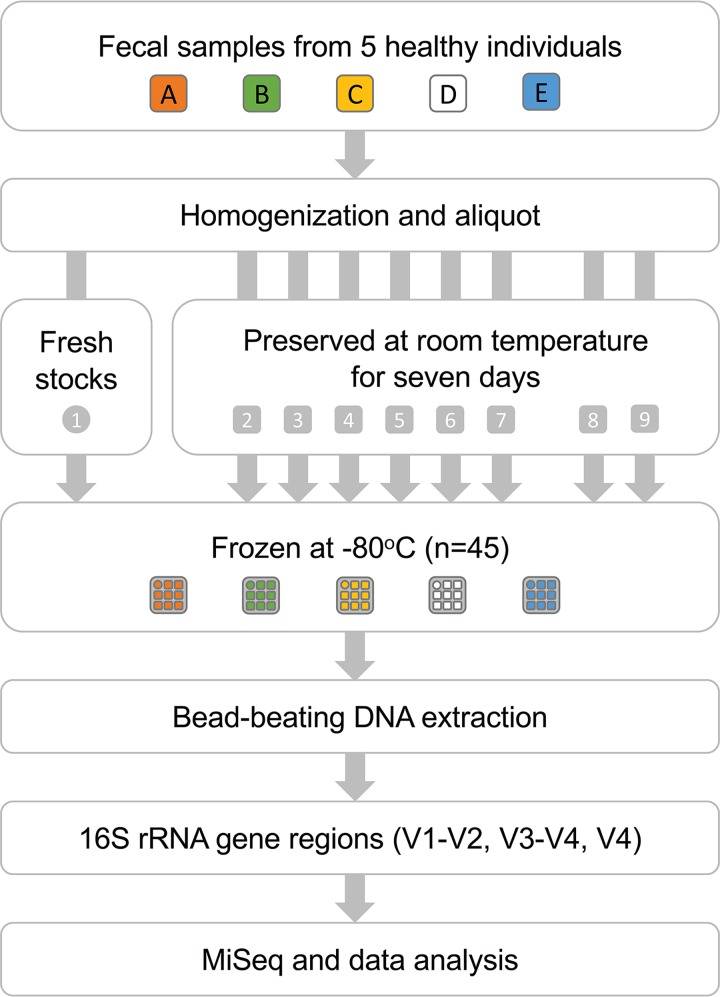
Workflow of the study design. Nine stool sample aliquots from each of the five donors were subjected to different preservatives and incubated at room temperature for 7 days to compare shifts in gut microbial community composition.

Five healthy individuals provided fresh stool samples (Fig. 1; see also Table S1 in the supplemental material). For each subject, one subset of sample was immediately frozen at −80°C; eight aliquots were stored in different solutions, including six preservatives (Norgen, OMNI, RNAlater, CURNA, HEMA, and Shield), phosphate-buffered saline (PBS) solution, and Cary-Blair (CB) transport medium (Table 1). Following 7 days of preservation at room temperature, all aliquots were stored at −80°C without a freeze-thaw cycle until DNA extraction. Total DNA, including −80°C “gold standards,” was extracted with a bead-beating method and amplicon sequenced on an Illumina MiSeq platform by targeting three hypervariable regions of the bacterial 16S rRNA gene (V1-V2, V3-V4, and V4) (Table 2). No obvious difference in quality of extracted DNA for PCR amplification was observed between subjects or preservatives, as visualized on the agarose gel with similar band brightness (data not shown).

**TABLE 1:** List of DNA/RNA preservation methods used in this study

Name^*a*^	Description	Producer	Catalogue no.
1_Standard	Stock immediately frozen at −80°C		
2_Norgen	Stool nucleic acid preservative	Norgen Biotek	63700
3_OMNI	OMNIgene.GUT	DNA Genotek	OMR-200
4_RNAlater	RNAlater RNA stabilization reagent	Qiagen	76106
5_CURNA	Homemade RNA preservative		
6_HEMA	HEMAgene.BUFFY COAT	DNA Genotek	HG-BCD-50
7_Shield	DNA/RNA shield transport medium	Zymo Research	R1100-50
8_PBS	Phosphate-buffered saline	Thermo Fisher	10010023
9_CB	Cary-Blair transport medium	Puritan	CB-206

a The original stocks (1_Standard) were immediately frozen at −80°C. All samples preserved in different solutions (samples 2 to 9) were incubated at room temperature for 7 days and then frozen at −80°C.

**TABLE 2:** 16S rRNA gene PCR primer sequences used in this study

Region	Amplicon size (bp)^*a*^	Name	Direction	Primer sequence (5′–3′)
V1-V2	349	27F-YM	Forward	AGAGTTTGATYMTGGCTCAG
		338R	Reverse	TGCTGCCTCCCGTAGGAGT
V3-V4	466	341F	Forward	CCTACGGGNGGCWGCAG
		806RB	Reverse	GGACTACNVGGGTWTCTAAT
V4	292	515F	Forward	GTGYCAGCMGCCGCGGTA
		806RB	Reverse	GGACTACNVGGGTWTCTAAT

a Mean amplicon size shown for hypervariable region amplified plus PCR primers.

10.1128/mSystems.00271-18.7 TABLE S1 Time intervals of stool sample collection and processing. Download Table S1, XLSX file, 0.01 MB. Copyright © 2019 Chen et al. 2019 Chen et al. This content is distributed under the terms of the Creative Commons Attribution 4.0 International license.

### Variation in gut microbial communities without preservative at room temperature.

**FIG 2:**
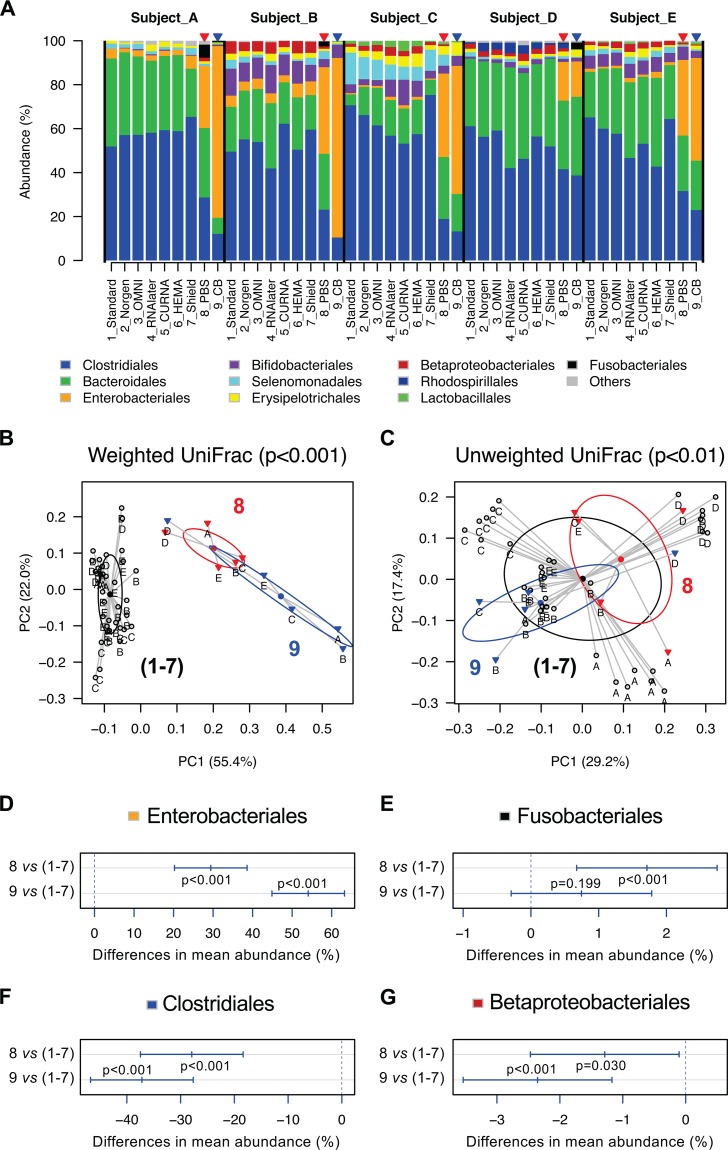
Community shift in nonpreserved stool samples in PBS or Cary-Blair transport medium observed by 16S rRNA gene V3-V4 amplicon sequencing. (A) Bar chart showing the proportion of the bacterial order. Filled triangles in red and blue represent nonpreserved samples in PBS (samples 8) and Cary-Blair transport medium (samples 9), respectively. The bacterial orders grouped into “Others” include *Coriobacteriales*, *Desulfovibrionales*, *Pasteurellales*, *Synergistales*, *Victivallales*, and *Bacillales*. (B) Principal-coordinate plot of weighted UniFrac distances discriminated nonpreserved stool samples from other aliquots in different preservatives (Table 1 gives detailed preservation methods for samples 1 to 7). (C) Principal-coordinate plot of unweighted UniFrac distances discriminated nonpreserved stool samples from other aliquots in different preservatives. (D to G) A pairwise Tukey HSD *post hoc* test shows an overgrowth of the relative abundance of *Enterobacteriales* and *Fusobacteriales* and a reduction of relative abundance of *Clostridiales* and *Betaproteobacteriales* in nonpreserved stool samples.

We first used sequences representing the V3-V4 region of the 16S rRNA gene to assess gut microbiota profile since this region provides the longest read length (Table 2). A total of 121,532 high-quality sequence reads for 45 samples (mean ± SD of 2,700 ± 667) was obtained, clustering into 308 amplicon sequence variants (ASVs). These ASVs represented 105 bacterial taxa at genera or higher levels, while 64 were detected at ≥1% relative abundance in at least one sample. The gut microbial community assigned at order level is displayed in Fig. 2A. As expected, the nonpreserved stool samples stored in PBS (8_PBS) and Cary-Blair transport medium (9_CB) at room temperature for 7 days had significant changes in microbiota composition compared to −80°C standards and preserved stocks (samples 1 to 7), as discriminated by a principal-coordinate analysis (PCoA) using either weighted or unweighted UniFrac distances (Fig. 2B and C). Using the Adonis function in R’s package Vegan, however, a permutational multivariate analysis of variance (PERMANOVA) based on weighted UniFrac distances (df = 2, *R*^2^ = 0.504, pseudo-*F* = 21.346, *P* < 0.001) found a higher variability in microbial community composition than unweighted UniFrac distances (df = 2, *R*^2^ = 0.093, pseudo-*F* = 2.160, *P* < 0.01) in separating nonpreserved samples from preserved ones, indicating that the sample clustering was driven more by the proportion of microbial community members than the presence/absence of bacterial taxa. For example, a significant increase of the relative abundance of *Enterobacteriales* (Fig. 2D) and *Fusobacteriales* (Fig. 2E) and a reduction of *Clostridiales* (Fig. 2F) and *Betaproteobacteriales* (Fig. 2G) were observed in nonpreserved gut samples (Table S2).

10.1128/mSystems.00271-18.8 TABLE S2 Pairwise Tukey HSD *post hoc* and nonparametric Mann-Whitney tests showing the different abundances of bacterial genera between nonpreserved and preserved stool samples based on 16S rRNA gene V3-V4 amplicon sequencing. 1 to 7, seven preservation methods; 8, PBS; 9, Cary-Blair transport medium. Statistical tests where *P* < 0.05 are highlighted in bold. Download Table S2, XLSX file, 0.01 MB. Copyright © 2019 Chen et al. 2019 Chen et al. This content is distributed under the terms of the Creative Commons Attribution 4.0 International license.

### Variation in gut microbial communities stored in preservatives.

**FIG 3:**
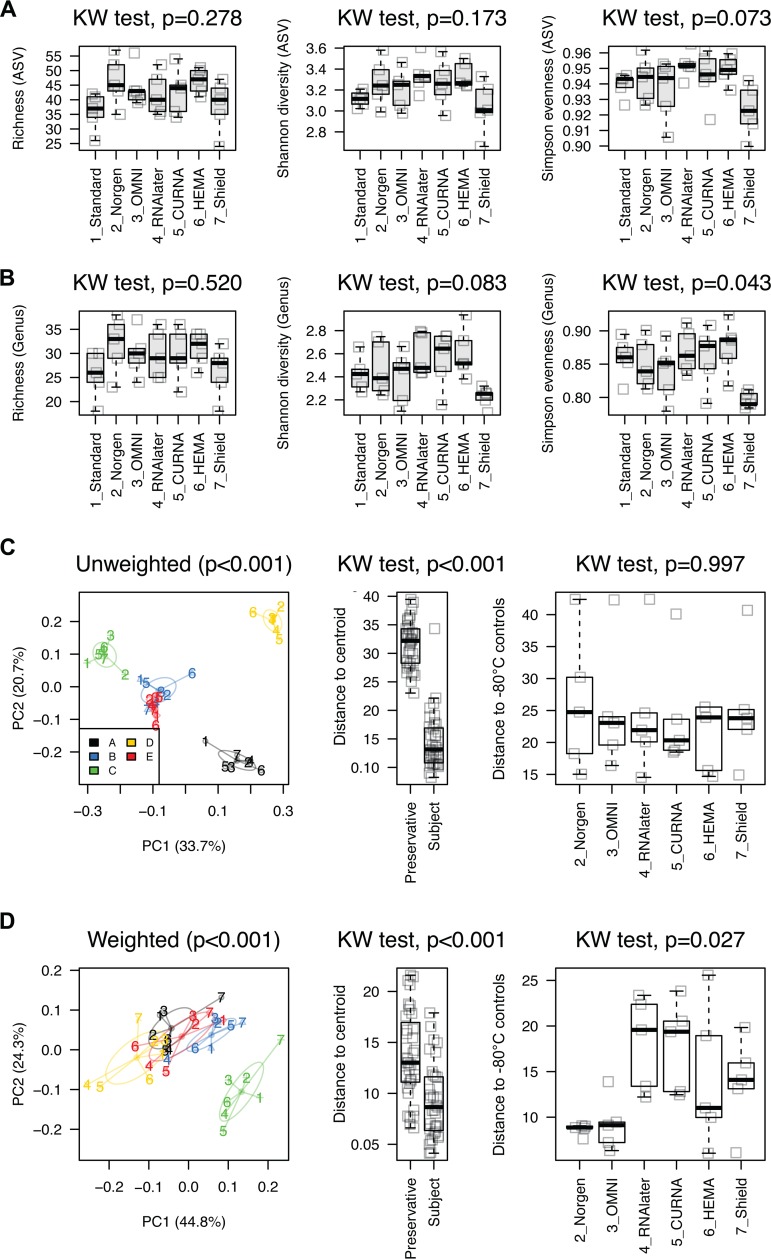
Comparison of gut microbiota alpha diversity in different preservatives based on 16S rRNA gene V3-V4 amplicon sequencing. Alpha diversity analysis on amplicon sequence variant (ASV) (A) and genus (B) levels. Principal-coordinate plot using unweighted (C) and weighted (D) UniFrac distances, with sample clustering by individual subjects. The PC1 and PC2 axes represent the first two principal coordinates. KW test, Kruskal-Wallis test.

Next, we refined the ASV table by excluding samples in PBS solution and Cary-Blair transport medium to measure the variation of six preservatives in retaining gut microbiota composition with reference to immediate freezing at −80°C as standards. Overall, the microbial community of the surveyed stool samples was mainly dominated by *Faecalibacterium* (mean relative abundance of 23.4%) and *Bacteroides* (22.2%), followed by *Roseburia* (7.3%) (Table S3). The alpha diversity analyses at either ASV or genus level did not differ significantly between −80°C standards and preserved samples (Fig. 3A and B), except that aliquots stored in Shield appeared relatively lower in Shannon diversity (mean of 3.0 and 2.2, respectively) and Simpson evenness (mean of 0.9 and 0.8, respectively). We found significant changes of the alpha diversities of gut microbial community across subjects (Kruskal-Wallis test, *P* < 0.029) (Fig. S1).

10.1128/mSystems.00271-18.2 FIG S1 Comparison of gut microbiota diversity between individual subjects based on 16S rRNA gene V3-V4 amplicon sequencing. Download FIG S1, TIF file, 0.7 MB. Copyright © 2019 Chen et al. 2019 Chen et al. This content is distributed under the terms of the Creative Commons Attribution 4.0 International license.

10.1128/mSystems.00271-18.9 TABLE S3 Relative abundances of gut microbiota in different preservatives. Pairwise Tukey HSD *post hoc* test and nonparametric Wilcoxon signed-rank test were used to detect statistical significance. The proportions of each bacterial taxon were summarized at the genus level or above. Download Table S3, XLSX file, 0.2 MB. Copyright © 2019 Chen et al. 2019 Chen et al. This content is distributed under the terms of the Creative Commons Attribution 4.0 International license.

A PERMANOVA using weighted UniFrac distances indicated that both subject groups (df = 4, *R*^2^ = 0.632, pseudo-*F* = 26.134, *P* < 0.001) and preservation conditions (df = 6, *R*^2^ = 0.223, pseudo-*F* = 6.157, *P* < 0.001) contributed to the differences in microbiota composition, although the majority of variability was from differences between subjects rather than the lower impact of storage conditions (63.2% versus 22.3%). The variation was statistically attributed to subjects (df = 4, *R*^2^ = 0.779, pseudo-*F* = 28.034, *P* < 0.001) but not preservatives (df = 6, *R*^2^ = 0.055, pseudo-*F* = 1.309, *P* = 0.118) based on unweighted UniFrac distances. In line with these observations, the principal-coordinate analysis supported that microbial variation was mainly associated with differences between individuals (Fig. 3C and D). In order to examine the small effects of variation by preservation methods, we subsequently calculated the pairwise distances between −80°C standards and preserved samples collected from the same subjects using weighted UniFrac distances, since unweighted UniFrac distances did not reveal statistical significance (*P* = 0.997) (Fig. 3C). As shown in Fig. 3D (weighted), samples stored in various preservatives exhibited diversified microbial community shifts relative to −80°C standards (intrasubject distance to standard of 0.06 to 0.26) (*P* = 0.027), suggesting that all preservation methods contributed impacts on the variation in microbiota profiling. We found, however, that two preservatives (Norgen and OMNI) showed the least shift in community composition (mean distance to the standards of 0.09 and 0.09, respectively) while RNAlater had the highest impact (mean distance to the standards of 0.18). Consistent with the pairwise distance comparison, the relative abundance of *Bacteroides* and *Ruminococcaceae* UCG-002 increased significantly in samples stored in RNAlater compared to −80°C standards (Tukey honest significant difference [HSD] *post hoc* test, *P* = 0.0499 and *P* = 0.0408, respectively) (Table S3). Similarly, an increase of the relative abundance of *Faecalibacterium* from samples stored in Shield (*P* = 0.0239) and a decrease of the relative abundance of *Lachnospiraceae* (Eubacterium eligens group) in OMNI (*P* = 0.0461) relative to −80°C standards were observed.

### Impact of targeting different regions of the 16S rRNA gene in profiling gut microbiota.

**FIG 4:**
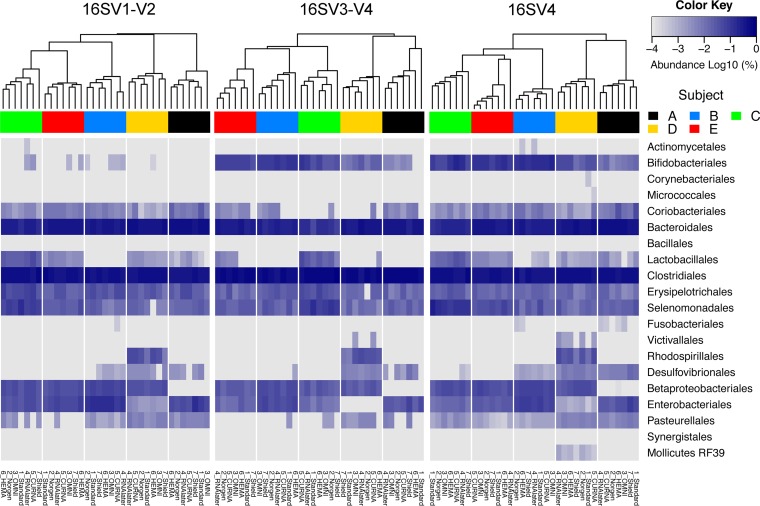
Heat map comparing the proportional abundances of gut microbiota summarized at the order level between 16S rRNA gene primer pairs. Each column represents individuals with the proportional abundance on a base 10 logarithmic scale, with 0 (a log_10_ of 100%) in black and −4 (a log_10_ of 0.01%) in light silver. Each row represents the bacterial order. The clustering of subjects at the top is based on the weighted GUniFrac distance.

Previous work has suggested that targeting different variable regions of the 16S rRNA gene may generate different microbial community profiles (20). In this study, we compared the different performances of primer pairs targeting three hypervariable regions of bacterial 16S rRNA gene (V1-V2, V3-V4, and V4) in profiling gut microbiota composition. Overall, 254,363 and 420,622 high-quality sequence reads (mean ± SD of 7,267 ± 1,139 and 12,017 ± 3,799) were obtained with V1-V2 and V4 primer pairs, respectively. When analyses on the divergences of preservation methods in revealing gut microbial community were repeated, the sequences generated by V1-V2 and V4 primer pairs (Fig. 4; Fig. S2 and S3) showed similar results as that of V3-V4 reads. The surveyed stool samples mainly clustered by subjects. Although variations in microbiota composition were observed for samples in all preservatives tested, the effects were relatively small (PERMANOVAs using weighted UniFrac distances: V1-V2, *R*^2^ = 0.163, *P* < 0.001; V4, *R*^2^ = 0.167, *P* < 0.001) compared to the high heterogeneity between individuals (V1-V2, *R*^2^ = 0.709, *P* < 0.001; V4, *R*^2^ = 0.709, *P* < 0.001). Samples in Norgen preservative consistently showed the least shift in community composition relative to −80°C standards. Meantime, the relative abundance of *Faecalibacterium* in samples stored in Shield was significantly higher, as revealed by all three 16S rRNA gene region primer pairs (Table S3).

10.1128/mSystems.00271-18.3 FIG S2 Comparison of gut microbiota diversities from samples in different preservatives observed using 16S rRNA gene V1-V2 amplicon sequencing. Download FIG S2, TIF file, 1.4 MB. Copyright © 2019 Chen et al. 2019 Chen et al. This content is distributed under the terms of the Creative Commons Attribution 4.0 International license.

10.1128/mSystems.00271-18.4 FIG S3 Comparison of gut microbiota diversities from samples in different preservatives observed using 16S rRNA gene V4 amplicon sequencing. Download FIG S3, TIF file, 1.4 MB. Copyright © 2019 Chen et al. 2019 Chen et al. This content is distributed under the terms of the Creative Commons Attribution 4.0 International license.

**FIG 5:**
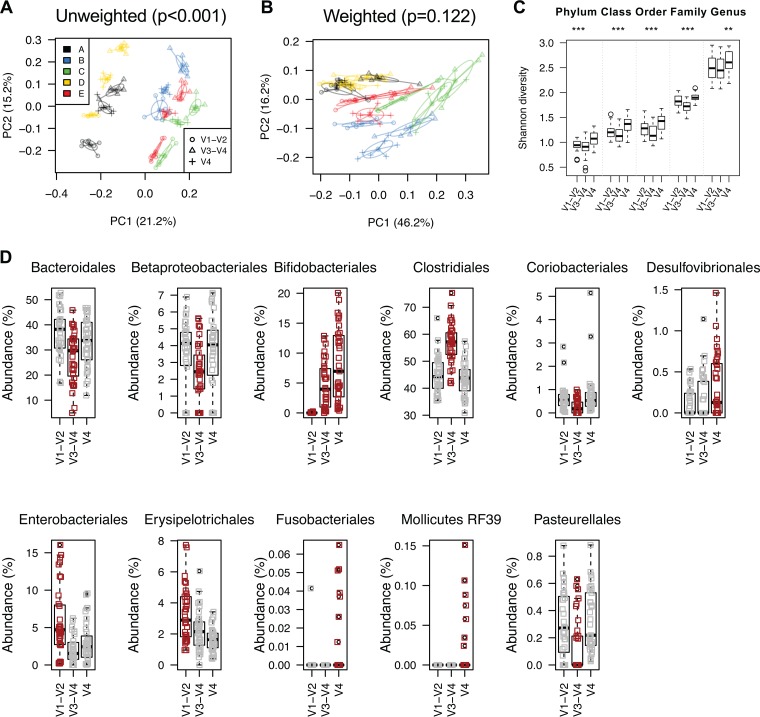
(A and B) Unweighted (A) and weighted (B) UniFrac clustering of three 16S rRNA hypervariable regions using fragment insertion SATé-enabled phylogenetic placement (SEPP) technique. (C) Box plot of the Shannon diversity indices between 16S rRNA V1-V2, V3-V4, and V4 regions. Statistical significance was calculated using Kruskal-Wallis test. (D) Relative abundances (at the order level) of gut microbiota showing statistically significant differences between 16S rRNA V1-V2, V3-V4, and V4 regions. Taxa in red show statistical significance according to nonparametric Wilcoxon signed-rank test (*P* < 0.05) (see details in Table S4).

However, the choice of primers targeting different hypervariable regions of the bacterial 16S rRNA gene may specifically impact microbial community profile and bacterial taxon assessment. When all three region reads were combined for principal-coordinate analyses and PERMANOVAs using fragment insertion SATé-enabled phylogenetic placement (SEPP) technique, we found strong bias of gene regions in sample clustering based on either unweighted (df = 2, *R*^2^ = 0.218, pseudo-*F* = 42.239, *P* < 0.001) or weighted (df = 2, *R*^2^ = 0.197, pseudo-*F* = 51.940, *P* < 0.001) UniFrac distances, although the majority of variability was consistently from differences between subjects (unweighted, df = 4, *R*^2^ = 0.527, pseudo-*F* = 51.073, *P* < 0.001; weighted, df = 4, *R*^2^ = 0.507, pseudo-*F* = 66.863, *P* < 0.001) (Fig. 5A and B). The impact of preservation methods on microbial community shifts was relatively small (unweighted, df = 6, *R*^2^ = 0.018, pseudo-*F* = 1.156, *P* = 0.198; weighted, df = 6, *R*^2^ = 0.122, pseudo-*F* = 10.707, *P* < 0.001). We also found that the V4 prime pair showed a higher Shannon diversity for gut microbiota profile than did other primer sets (Fig. 5C), in line with the higher levels of alpha diversity with rarefied V4 region reads (at genus level and above) (Fig. S4). The relative abundance of 11 bacterial orders was significantly variable among reads generated by different primers (Fig. 5D; Table S4). For example, the commonly used V1-V2 primer pair failed to detect the majority of *Bifidobacteriales* (mean relative abundance of 0.03% versus 6.40%, *P* < 0.001) but revealed more *Enterobacteriales* (5.69% versus 2.57%, *P* < 0.001) and *Erysipelotrichales* (3.2% versus 2.0%, *P* < 0.001) compared to another two primer sets. The V3-V4 primers showed the highest abundance of *Clostridiales* (56.69% versus 44.12%, *P* < 0.001), which likely led to the apparent decrease of *Bacteroidales* (26.91% versus 34.97%, *P* < 0.001), *Betaproteobacteriales* (2.45% versus 3.58%, *P* < 0.001), *Coriobacteriales* (0.26% versus 0.71%, *P* < 0.001), and *Pasteurellales* (0.13% versus 0.31%, *P* < 0.001). *Mollicutes* RF39 was amplified only by the V4 primer set, although the relative abundance of this bacterial group was extremely low in the surveyed stool samples.

10.1128/mSystems.00271-18.5 FIG S4 Rarefaction curves of species richness (A) and Shannon diversity (B) of gut microbiota summarized at different taxonomy levels. Download FIG S4, TIF file, 1.3 MB. Copyright © 2019 Chen et al. 2019 Chen et al. This content is distributed under the terms of the Creative Commons Attribution 4.0 International license.

10.1128/mSystems.00271-18.10 TABLE S4 Relative abundances of gut microbiota that were differentially enriched or depleted according to the choice of 16S rRNA gene primer pairs. The proportions of each bacterial taxon were summarized at the order level. Download Table S4, XLSX file, 0.02 MB. Copyright © 2019 Chen et al. 2019 Chen et al. This content is distributed under the terms of the Creative Commons Attribution 4.0 International license.

### Sequence variation of primer-binding sites.

**FIG 6:**
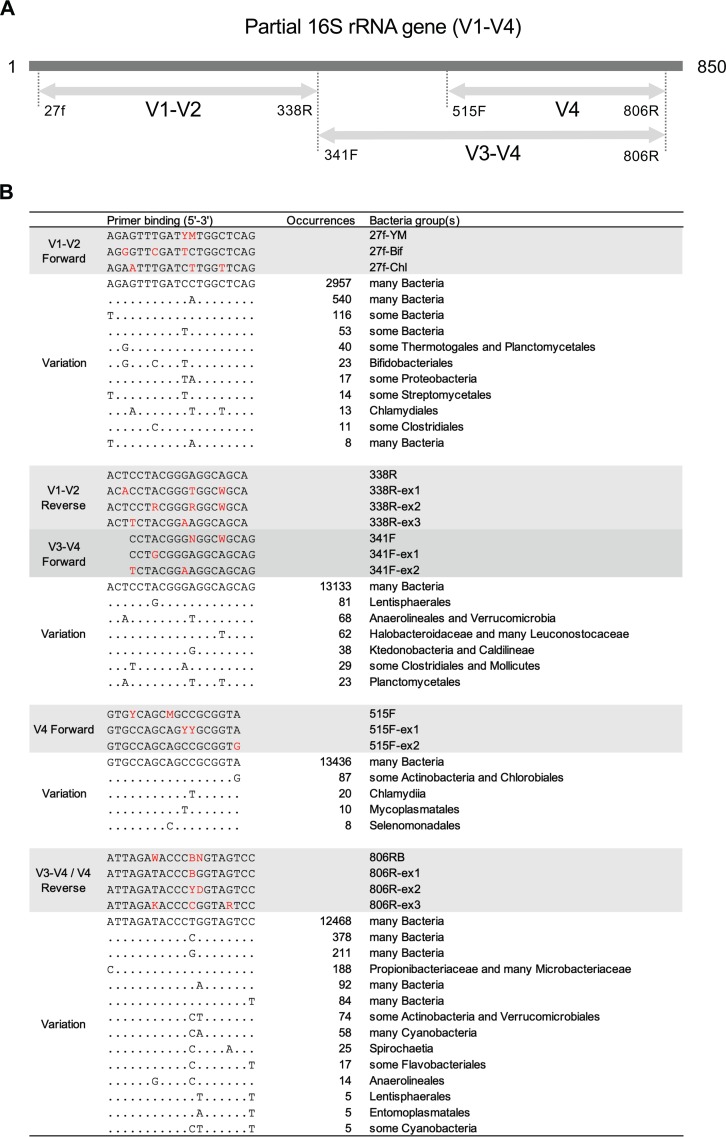
Sequence variation of 16S rRNA gene primer-binding sites. (A) Schematic of the 16S rRNA gene regions and primer pairs. (B) Occurrences of the most commonly observed sequences of primer-binding sites and the primer formulations that they may match. Nucleotide sites identical to the most common consensus sequence (the first listed) are represented as dots. The main nucleotide changes of primer sequences from the most common consensus sequence are highlighted in red.

In order to characterize the occurrences of sequence variations at the primer-binding sites used in this study, we refined an NCBI bacterial 16S rRNA reference database (a total of 18,773 complete or near-complete sequences) using a threshold of 99% similarity and extracted sequence sets matching each primer-binding site. The most common sequence variations are shown in Fig. 6, with the corresponding dominant bacterial group(s) in which the sequence variations are observed. It is noted that the V1-V2 forward primer-binding site sequences in most *Bifidobacteriales* and *Chlamydiales* 16S rRNA genes were not precisely accommodated by the commonly used degenerate 27f primers, differing at two and three positions from the best-matching 27f-YM primer, respectively. Similarly, the nondegenerate form of the V1-V2 reverse primer used in this study (338R) may have low sensitivity in detecting *Planctomycetales*, for which three mispairings were identified. The V1-V2 reverse primer (338R) and V3-V4 forward primer (341F) showed mismatches with *Mollicutes* RF39 16S rRNA genes in two positions, which could confer a low efficiency in detecting this group of bacteria (Fig. 5D). Optimization of primer pools to minimize the mispairing at primer-binding sites is proposed (Fig. 6B).

### Bacterial culture of stool samples in different preservatives.

To test whether the surveyed preservatives are able to rend microbes noninfectious, stool suspensions preserved in each solution over time were cultured for bacteria under both aerobic and anaerobic conditions (Fig. S5). We found that Norgen, OMNI, HEMA, and Shield preservatives immediately and completely prevented the growth of gut bacteria, allowing the resulting samples to be handled safely. In contrast, RNAlater and CURNA, two preservation solutions designed for RNA samples, as well as the PBS and Cary-Blair transport medium, showed no effect in inactivating bacteria.

10.1128/mSystems.00271-18.6 FIG S5 Aerobic and anaerobic bacterial culture for stool samples with different preservation methods. The *y* axis represents log_10_ CFU counts. Download FIG S5, TIF file, 0.9 MB. Copyright © 2019 Chen et al. 2019 Chen et al. This content is distributed under the terms of the Creative Commons Attribution 4.0 International license.

## DISCUSSION

Microbiota studies have been quickly switched to large-scale sample collection to characterize the subtle differences in microbial communities in relation to human health and diseases. Although immediate freezing at −20°C or below has been considered the “gold standard” for microbiota preservation, this approach is not feasible for many studies, especially field sampling from remote areas or self-collected samples requiring shipment to the laboratory without cold chain transport. In such cases, a proper preservation method to minimize microbiota composition shift to reduce bias and to inactivate infectious agents for samples to be handled safely becomes critical. Hence, we evaluated the effects of multiple preservation solutions and 16S rRNA gene hypervariable regions in revealing gut microbiota composition. Our data showed that the profile of gut microbiota can change dramatically over a course of 7 days at room temperature in the absence of protection of preservation. The use of a proper preservative without cold chain, in contrast, provides satisfactory prevention of community shifts. The effects of these preservatives were small compared to intersubject variations; however, notable changes in microbiota composition were observed. Nevertheless, we found that two preservatives, Norgen and OMNI, showed the smallest microbial community shifts, and both efficiently inhibited the growth of aerobic and anaerobic bacteria, suggesting they may serve as useful solutions for stool sample transportation and storage. Recent studies have reported that OMNIgene.GUT (here called OMNI) is able to stabilize DNA at room temperature for up to 14 days, with little difference in microbiota composition by 16S rRNA gene sequencing (13, 15, 19, 21). RNAlater, a commonly used preservative for RNA samples, on the other hand, exhibited larger community shift in bacterial relative abundance at room temperature for 7 days, consistent with previous reports that the microbial community in RNAlater may lose stability at longer time frames when maintained at ambient temperatures (12, 15, 16, 22). However, under cold conditions (e.g., 4°C or −20°C) or for a short period of room-temperature storage (e.g., within same or another day), it performed equally well as other storage conditions, such as 95% ethanol, FTA cards, and OMNIgene.GUT (21, 23, 24). Interestingly, RNAlater and CURNA (with similar components as RNAlater) do not prevent bacterial activity, highlighting themselves as potential alternatives for microbial sample collection when live bacterial culture is necessary. According to the manufacturer’s instructions, HEMA was initially developed for the stabilization of buffy coat samples and there was no previous report evaluating its use as preservative for samples intended for microbiota studies. Shield performed well in microbiota preservation and disinfection but consistently showed increased relative abundance of *Faecalibacterium* regardless of the choice of 16S rRNA gene primer pairs. We recommend the use of Norgen or OMNI for microbial sample preservation and caution against RNAlater for long-term storage at ambient temperatures.

Choosing primers to target certain regions of the 16S rRNA gene for bacterial taxonomic profiling is another difficult challenge, and the comparability of primers with microbes from various communities requires attention (25, 26). The V1 to V4 region, for example, is more divergent than V4 to V7 and can provide a higher resolution in bacterial clustering for stool samples (20, 27). In this work, we compared the performance of three commonly used primer pairs targeting the 16S rRNA gene V1-V2, V3-V4, and V4 regions in profiling gut microbial community and found a higher alpha diversity and richness revealed by V4 primers. Our results also indicate an immense impact of primers with regard to amplification of bacterial taxa. Close examination of primer-binding sites revealed several sequence variations within cohesive phylogenetic groups (e.g., *Bifidobacteriales*) that are not accommodated by the commonly used 27f primer sequence. In such cases, the use of pooled primers to minimize mismatches with this bacterial group will provide an effective approach for revealing the true bacterial diversity (26, 28, 29). Knowledge of how primer pairs differentially amplify bacterial taxa is important for study design and relating results to prior studies. Alternatively, the quickly developing next-generation sequencing platforms, such as PacBio and Nanopore, may facilitate the characterization of full-length bacterial 16S rRNA genes and allow global comparisons of microbiome studies across sample types and gene regions (30–32).

This study has its strengths and limitations. We expand the current understanding of the performance of several DNA and RNA preservation solutions, including CURNA and HEMA, to our knowledge being evaluated for the first time, in retaining gut microbiota composition. The data provide a useful reference for knowing the biases introduced by storage conditions. We have expanded the knowledge of bactericidal ability of the surveyed preservatives that may provide alternatives in study design considering the potential of infectious agents and bacterial culture. We have also identified the significant difference of 16S rRNA gene primer pairs in charactering bacterial taxa and emphasized the importance of a consistent methodology for large epidemiological studies. Nevertheless, one of the shortcomings of this study is the small sample size and the conserved sample source (5 subjects who were all male, young, and healthy), which may underestimate the impact of preservation solutions and primer pairs in charactering the diversity of gut microbiota. Further methodological comparison is needed to expand to other settings, including patient groups and population-based epidemiological studies. Only sequencing-based evaluation was performed in this work, while the concordance of preservation solutions in other omics studies, such as proteomics and metabolomics, may be largely variable. For example, RNAlater was reported to be not feasible for metabolomics measures probably due to its high sodium sulfate content, making the preserved samples incompatible with metabolomics platforms (22, 33). Last, more robust qualitative and quantitative testing under variable conditions, such as extreme temperatures, increased freeze-thaw cycles, or longer storage periods, may provide a more comprehensive evaluation of the effectiveness and stabilization of storage methods at preserving microbial DNA quality and yield.

### Conclusions.

It is important for large-scale microbiome studies to accurately and consistently reveal microbial communities while minimizing external effects. Choices of preservation solution for transportation of stool samples at ambient temperature and of gene region for 16S rRNA gene amplicon sequencing are critical determinants impacting the observed microbial community profiling. We recommend the use of a proper preservative, such as Norgen or OMNI, that can minimize microbial community shift and efficiently inactivate bacterial growth, allowing the resulting samples to be handled and shipped safely and stably. Amplicon sequencing-based human microbiota studies are highly dependent on choice of primer pairs targeting different hypervariable regions of the bacterial 16S rRNA gene. Nevertheless, a consistent methodology is essential in minimizing batch biases to facilitate comparisons between data sets.

## MATERIALS AND METHODS

### Ethics approval.

This study was approved by the Joint Chinese University of Hong Kong-New Territories East Cluster Clinical Research Ethics Committee. All subjects recruited in this study were older than 18 years of age, and samples were anonymized without individual identifying information. Written informed consent was obtained from each participant.

### Stool specimen collection, processing, and DNA extraction.

Five healthy male subjects, aged between 23 and 28 years old, were recruited to provide fresh stool samples using a stool self-collection kit (see Table S1 in the supplemental material). Stools from these five donors were collected on different days and were transported to the laboratory in an icebox cooler at 4°C within 2 h without a freeze-thaw cycle. Upon receipt, approximately 5 g of sample was immediately mixed with 200 µl of sterile phosphate-buffered saline (PBS) and thoroughly homogenized for 1 min using a sterile cotton stick. One gram of the homogenized stool, serving as a standard, was transferred into a 2-ml sterile collection tube and immediately stored at −80°C until further DNA extraction. In parallel, eight stool aliquots, each weighing approximately 0.2 g, were transferred into 2-ml collection tubes preloaded with 1 ml of preservative (Norgen, OMNI, RNAlater, CURNA, HEMA, and Shield), PBS, or Cary-Blair transport medium, which is intended for preserving the viability of enteric bacteria (Table 1). Tubes were pulse vortexed for 5 s and placed on a laboratory bench at room temperature for 7 days. Then, stool aliquots were stored at −80°C without a freeze-thaw cycle for 28 to 73 days until DNA extraction (Fig. 1). The procedures of stool homogenization and aliquoting were performed in a biosafety cabinet and finished within 30 min.

DNA extraction was performed in accordance with the Earth Microbiome Project protocol (34), with the bead-beating QIAamp PowerFecal DNA kit (Qiagen, USA). In detail, 100 µl of stool suspension from aliquots stored at −80°C was added to the PowerBead tubes for total DNA extraction according to the manufacturer’s protocol. An MP FastPrep-24 5G homogenizer, at a speed of 6.0 m/s for 45 s, was used for mechanical lysis. Approximately 0.2 g of stool standards was suspended in 1 ml of sterile PBS prior to DNA extraction. The DNA samples were eluted in 100 µl elution buffer (pH 8.0) and stored at −20°C. DNA concentrations were measured using a NanoDrop 2000 (Thermo Scientific, USA).

### 16S rRNA gene PCR amplification and sequencing.

We used three PCR primer pairs targeting the 16S rRNA gene V1-V2, V3-V4, and V4 regions, respectively, to profile gut microbiota compositions (Table 2) (35). A pair of dual 12-bp barcodes was indexed to each amplicon set through the forward and reverse primers modified from the Earth Microbiome Project protocol (34). Successful amplicons were pooled at approximately equal molar DNA concentrations, purified using the QIAquick gel extraction kit (Qiagen, USA), and sequenced on an Illumina MiSeq (Illumina, USA) at the Weill Cornell Medicine Genomics Resources Core Facility, New York, NY, USA, using paired-end 300-bp reads.

### Bioinformatics and biostatistics analysis.

Following demultiplexing, the QIIME2 (2018.8) package (https://qiime2.org) (36) was applied to assign Illumina short reads into amplicon sequence variant (ASV) and bacterial taxa tables. The detailed pipelines, including quality control, paired-end short reads merging, and dada2 denoising and clustering, can be found in the supplemental pipeline (Text S1). In order to combine reads from different 16S rRNA hypervariable regions for principal coordinates analysis, the fragment insertion SATé-enabled phylogenetic placement (SEPP) technique with default parameters was used (37). The SILVA 132 99% 16S rRNA gene reference database (https://www.arb-silva.de/download/archive/qiime) was used to assign bacterial taxonomic classification (38). Singleton reads were removed for statistical analysis. Compositions of microbiota communities were summarized by proportion at different taxonomy levels, including genus, family, order, class, and phylum ranks.

10.1128/mSystems.00271-18.1 TEXT S1 Commands of bioinformatics workflow with QIIME2 for 16S rRNA amplicon sequence analysis. Download Text S1, PDF file, 0.1 MB. Copyright © 2019 Chen et al. 2019 Chen et al. This content is distributed under the terms of the Creative Commons Attribution 4.0 International license.

In order to describe the diversity of observed bacterial taxa taking into consideration richness and abundance, Shannon and Simpson diversity indexes (39) were calculated. We assessed the alpha diversity using species richness and Shannon diversity at various rarefaction depths ranging from 1 to 6,001 reads (with steps of 250 reads) and found no significant difference of alpha diversity using rarefied data sets at depth of 1,000 reads per sample or higher (Fig. S4). In order to retain all samples for diversity analysis, we set 1,500 reads per sample as rarefaction depth to normalize the data for differences in sequence count. We used a UniFrac algorithm in the GUniFrac R package (40), with unweight or weight (alpha = 1) on abundant lineages to calculate pairwise Kantorovich-Rubinstein (KR) distances between samples. Differences in community composition were assessed using permutational multivariate analysis of variance (PERMANOVA) in the Vegan R package. Principal-coordinate analysis was performed to visualize associations between community composition and experimental factors. Comparisons of the relative abundances of characteristic ASVs between defined groups were performed using nonparametric Mann-Whitney Wilcoxon rank sum test (MWU), Wilcoxon signed rank test (WSR), Kruskal-Wallis test (KW), or Tukey’s honest significant difference (Tukey HSD) *post hoc* test. Statistical analyses and plotting were performed in R (3.4.0). A two-sided *P* value of <0.05 was considered statistically significant.

### Bacterial aerobic and anaerobic culture.

Approximately 0.2 g of homogenized fresh stool samples was transferred into 2-ml collection tubes preloaded with 1 ml of preservative (Norgen, OMNI, RNAlater, CURNA, HEMA, and Shield), PBS, or Cary-Blair transport medium. For each sample, 100-µl replicate suspensions were incubated at room temperature for 0 min, 10 min, 1 h, 5 h, 24 h, 48 h, and 96 h, respectively. Approximately 100 µl of 1:10-diluted stool suspensions in 0.85% saline solution was spread on a blood agar plate and a K1 agar plate for aerobic and anaerobic culture, respectively. Plates were incubated at 37°C for 48 h in a duplicate setting, and the number of CFU was counted immediately afterwards.

### Data availability.

All sequence data generated from this study were deposited in the Sequence Read Archive with accession number PRJNA470603.
