# Supplementary figures and images for: Impact of Preservation Method and 16S rRNA Hypervariable Region on Gut Microbiota Profiling
Source: mSystems. 2019 Feb 26;4(1):e00271-18. doi: 10.1128/mSystems.00271-18 (PMC6392095; doi:10.1128/mSystems.00271-18)

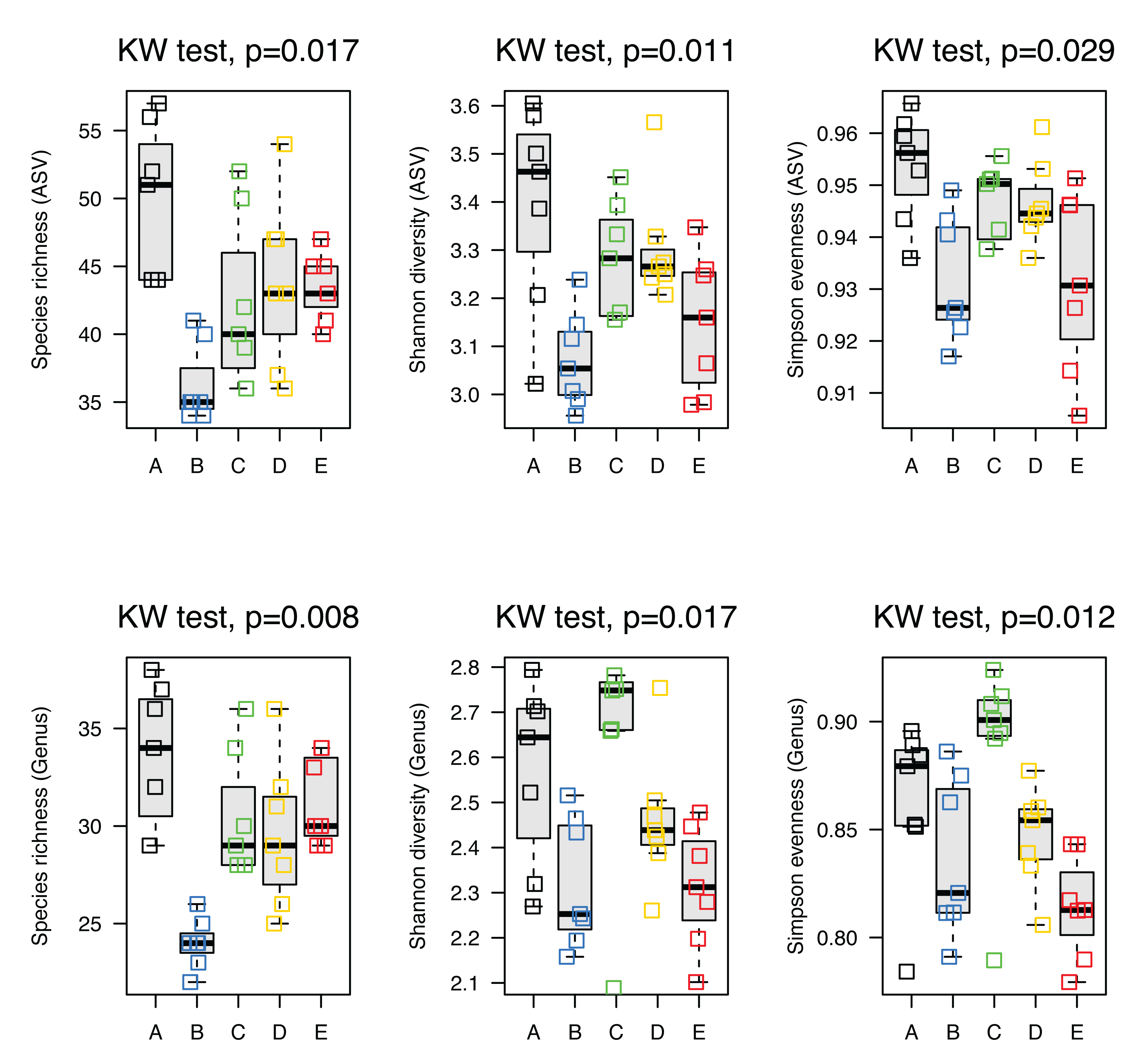

Supplement: FIG S1 [file mSystems.00271-18-sf001.tif]

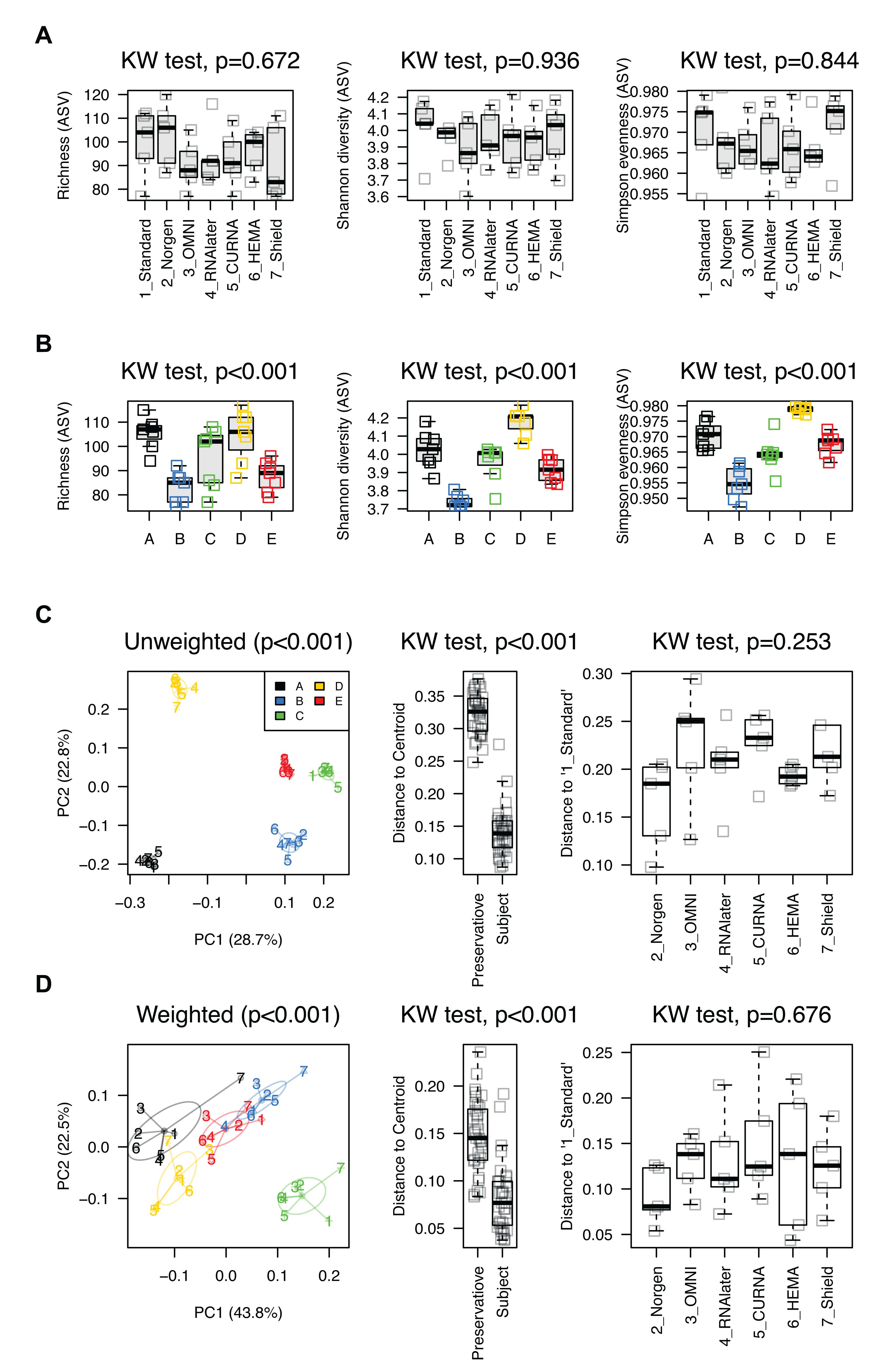

Supplement: FIG S2 [file mSystems.00271-18-sf002.tif]

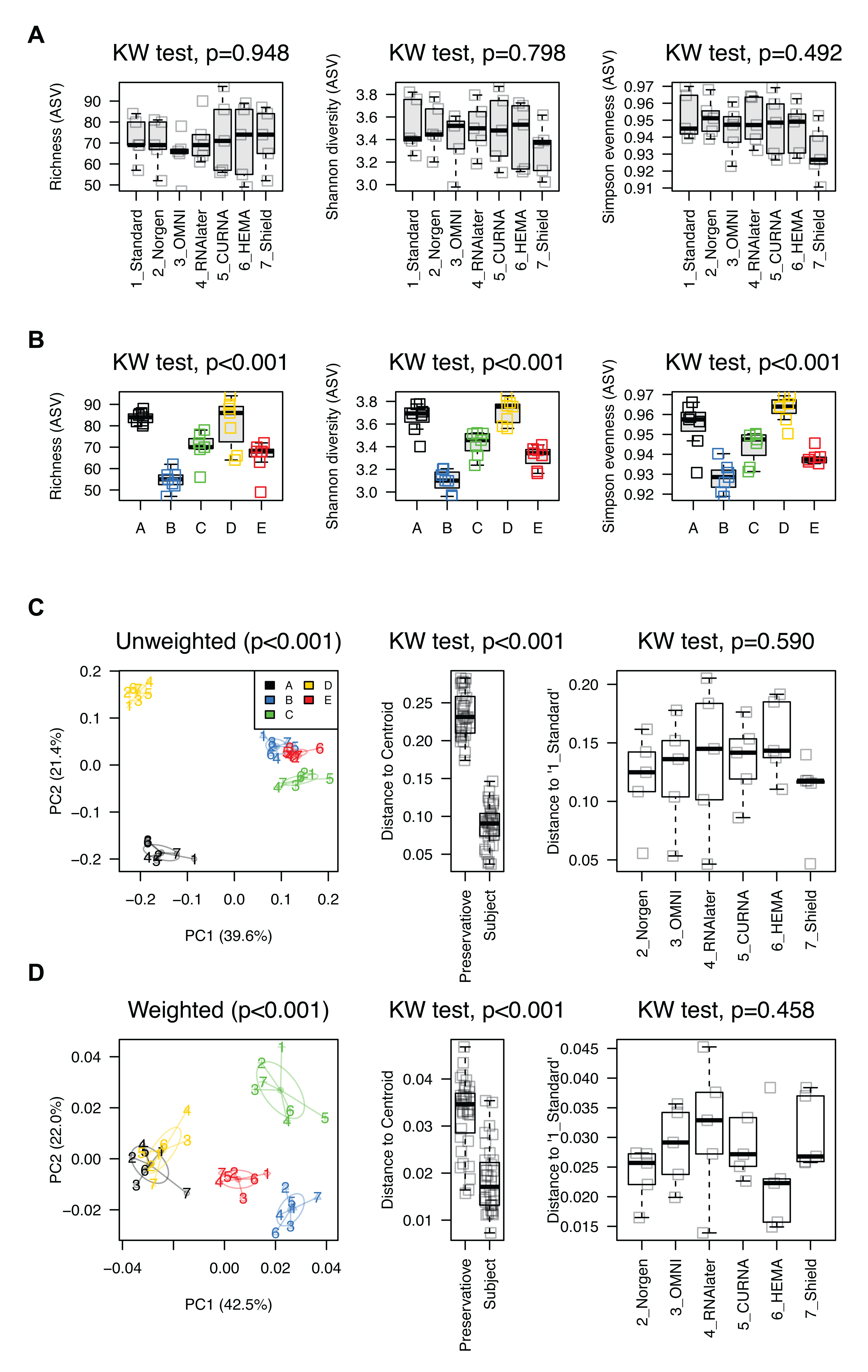

Supplement: FIG S3 [file mSystems.00271-18-sf003.tif]

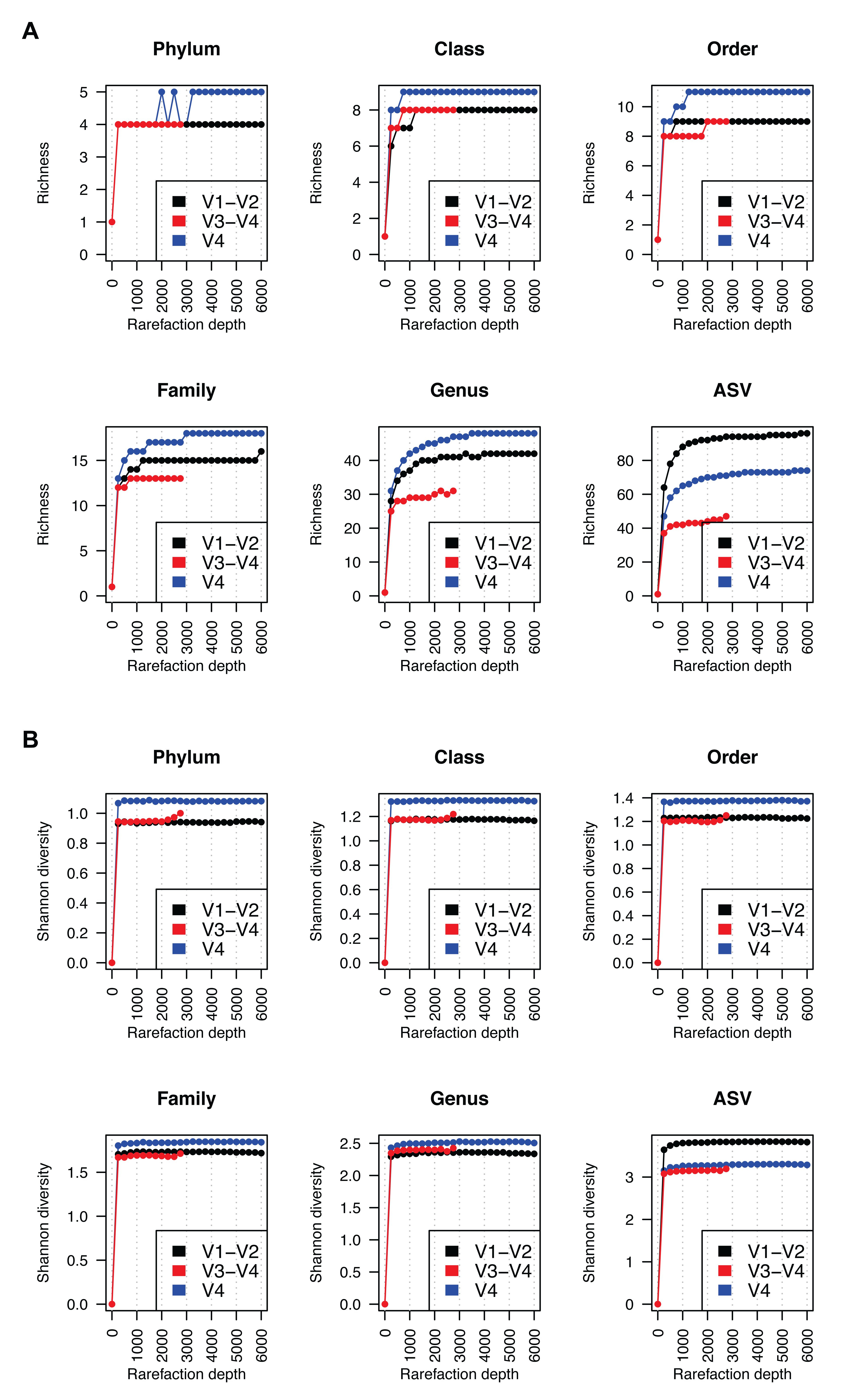

Supplement: FIG S4 [file mSystems.00271-18-sf004.tif]

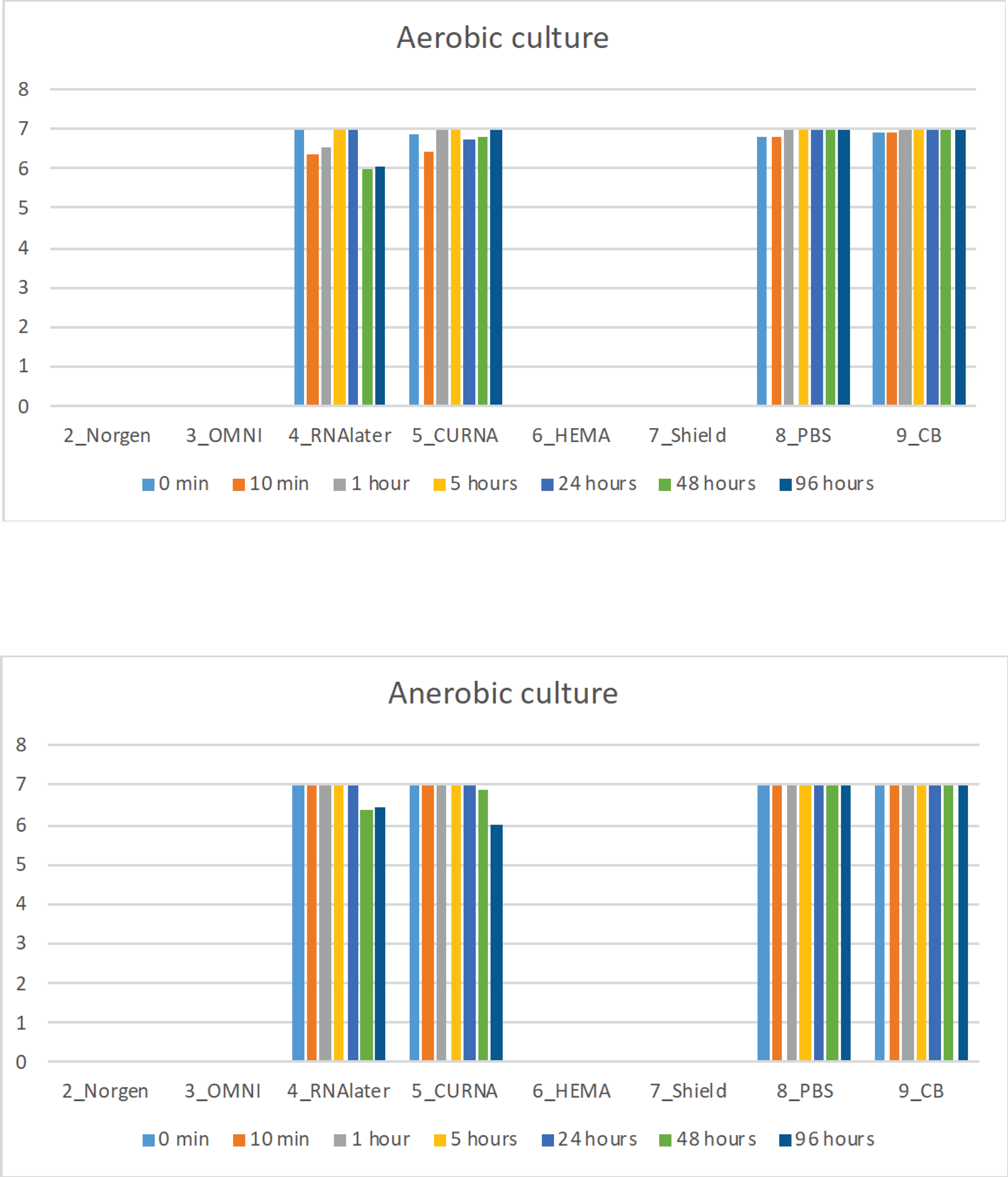

Supplement: FIG S5 [file mSystems.00271-18-sf005.tif]
