# Supplementary material for: Impact of Preservation Method and 16S rRNA Hypervariable Region on Gut Microbiota Profiling
Source: mSystems. 2019 Feb 26;4(1):e00271-18. doi: 10.1128/mSystems.00271-18 (PMC6392095; doi:10.1128/mSystems.00271-18)
Supplement: TEXT S1 [file mSystems.00271-18-s0001.pdf]

The following pipelines are applicable to the 16S V3-V4 dataset. Only key parameters for 16S V1-V2 and 16S V4 datasets are listed. All demultiplexed paired-end fastq sequences and metadata are available from SRA (PRJNA470603).

We used SILVA_132 99% reference database to train the feature classifiers. The full-length sequences is available from SILVA website (https://www.arb-silva.de/download/archive/qiime).

# loading qiime2-2018.8 environment

```
source activate qiime2-2018.8
```

# training 16S classifiers (SILVA_132_99)

```
qiime tools import \
     --type 'FeatureData[Sequence]' \
     --input-path 99/silva_132_99_16S.fna\
     --output-path 99_otus.qza

qiime tools import \
     --type 'FeatureData[Taxonomy]' \
     --source-format HeaderlessTSVTaxonomyFormat \
     --input-path 99/taxonomy_7_levels.txt \
     --output-path 99_taxonomy.qza
```

# extracting and training the SILVA 16SV3-V4 classifier

```
qiime feature-classifier extract-reads \
     --i-sequences 99_otus.qza \
     --p-f-primer CCTACGGGNGGCWGCAG \
     --p-r-primer GGACTACNVGGGTWTCTAAT \
     --p-trunc-len 466 \
     --o-reads ref-seqs-v34.qza

qiime feature-classifier fit-classifier-naive-bayes \
     --i-reference-reads ref-seqs-v34.qza \
     --i-reference-taxonomy 99_taxonomy.qza \
     --o-classifier silva132_v34_classifier.qza
```

# importing data

```
qiime tools import \
     --type 'SampleData[PairedEndSequencesWithQuality]' \
     --input-path /Folder_Containg_16SV3V4_Paired_End_Fastq/ \
     --input-format CasavaOneEightSingleLanePerSampleDirFmt \
     --output-path demux-paired-end-v34.qza
```

# denoising 16S V3-V4 data using dada2

```
qiime dada2 denoise-paired \
     --i-demultiplexed-seqs demux-paired-end-v34.qza \
     --p-trim-left-f 17 \
     --p-trunc-len-f 250 \
     --p-trim-left-r 20 \
     --p-trunc-len-r 250 \
     --o-table table-v34.qza \
     --o-representative-sequences rep-seqs-v34.qza \
     --p-n-threads 12 \
     --output-dir dada2-v34
```

# assigning taxonomy

```
qiime feature-classifier classify-sklearn \
     --i-classifier silva132_v34_classifier.qza \
     --i-reads rep-seqs-v34.qza \
     --o-classification taxonomy-v34.qza \
     --p-read-orientation 'same' \
     --p-n-jobs 12
```

# make denovo phylogeny of OTUs

```
qiime alignment mafft \
     --i-sequences rep-seqs-v34.qza \
     --o-alignment aligned-rep-seqs-v34.qza

qiime alignment mask \
     --i-alignment aligned-rep-seqs-v34.qza \
     --o-masked-alignment masked-aligned-rep-seqs-v34.qza

qiime phylogeny fasttree \
     --i-alignment masked-aligned-rep-seqs-v34.qza \
     --o-tree unrooted-tree-v34.qza

qiime phylogeny midpoint-root \
     --i-tree unrooted-tree-v34.qza \
     --o-rooted-tree rooted-tree-v34.qza
```

# exporting tree as a newick formatted file

```
qiime tools export \
      --input-path rooted-tree-v34.qza \
      --output-path exported-rooted-tree-v34
```

# exporting OTU feature table

```
qiime tools export \
      --input-path table-v34.qza \
      --output-path exported-otu-table-v34

biom convert \
      -i exported-otu-table-v34/feature-table.biom \
      -o exported-otu-table-v34/feature-table-v34.tsv \
      --to-tsv \
      --table-type "OTU table"
```

# collapsing feature tables by the taxonomy at the specific level (--p-level 6==genus, 5==family, 4==order, 3==class, 2==phylum)

```
qiime taxa collapse \
      --i-table table-v34.qza \
      --i-taxonomy taxonomy-v34.qza \
      --p-level 6 \
      --o-collapsed-table collapsed-table-v34-l6

qiime tools export \
      --input-path collapsed-table-v34-l6.qza \
      --output-path exported-collapsed-table-v34

biom convert \
      -i exported-collapsed-table-v34/feature-table.biom \
      -o exported-collapsed-table-v34/otu-table-v34-l6.tsv \
      --to-tsv \
      --table-type "OTU table"
```

# exporting taxonomy

```
qiime tools export \
      --input-path taxonomy-v34.qza \
      --output-path exported-taxonomy-v34
```

# exporting representative OTU sequences

```
qiime tools export \
      --input-path rep-seqs-v34.qza \
      --output-path exported-rep-seqs-v34
```

# extracting and training the SILVA V1-V2 classifier

```
qiime feature-classifier extract-reads \
     --i-sequences 99_otus.qza \
     --p-f-primer AGAGTTTGATYMTGGCTCAG \
     --p-r-primer TGCTGCCTCCCGTAGGAGT \
     --p-trunc-len 349 \
     --o-reads ref-seqs-v12.qza

qiime feature-classifier fit-classifier-naive-bayes \
     --i-reference-reads ref-seqs-v12.qza \
     --i-reference-taxonomy 99_taxonomy.qza \
     --o-classifier silva132_v12_classifier.qza
```

# extracting and training the SILVA V4 classifier

```
qiime feature-classifier extract-reads \
     --i-sequences 99_otus.qza \
     --p-f-primer GTGYCAGCMGCCGCGGTA \
     --p-r-primer GGACTACNVGGGTWTCTAAT \
     --p-trunc-len 292 \
     --o-reads ref-seqs-v4.qza

qiime feature-classifier fit-classifier-naive-bayes \
     --i-reference-reads ref-seqs-v4.qza \
     --i-reference-taxonomy 99_taxonomy.qza \
     --o-classifier silva132_v4_classifier.qza
```

# denoising 16S V1-V2 data using dada2

```
qiime dada2 denoise-paired \
     --i-demultiplexed-seqs demux-paired-end-v12.qza \
     --p-trim-left-f 20 \
     --p-trunc-len-f 200 \
     --p-trim-left-r 19 \
     --p-trunc-len-r 200 \
     --o-table table-v12.qza \
     --o-representative-sequences rep-seqs-v12.qza \
     --p-n-threads 12 \
     --output-dir dada2-v12
```

# denoising 16S V4 data using dada2

```
qiime dada2 denoise-paired \
     --i-demultiplexed-seqs demux-paired-end-v4.qza \
     --p-trim-left-f 18 \
     --p-trunc-len-f 175 \
     --p-trim-left-r 20 \
     --p-trunc-len-r 175 \
     --o-table table-v4.qza \
     --o-representative-sequences rep-seqs-v4.qza \
     --p-n-threads 12 \
     --output-dir dada2-v4
```

# merge data from different 16S regions using fragment insertion AKA SEPP

```
qiime feature-table merge \
     --i-tables table-v12.qza \
     --i-tables table-v34.qza \
     --i-tables table-v4.qza \
     --o-merged-table insertion-table.qza

qiime feature-table merge-taxa \
     --i-data taxonomy-v12.qza \
     --i-data taxonomy-v34.qza \
     --i-data taxonomy-v4.qza \
     --o-merged-data insertion-taxonomy.qza

qiime feature-table merge-seqs \
     --i-data rep-seqs-v12.qza \
     --i-data rep-seqs-v34.qza \
     --i-data rep-seqs-v4.qza \
     --o-merged-data insertion-rep-seqs.qza

qiime fragment-insertion sepp \
     --i-representative-sequences insertion-rep-seqs.qza \
     --p-threads 12 \
     --o-tree insertion-tree.qza \
     --o-placements insertion-placements.qza
```

# Use ‘insertion-tree.qza’ for all downstream phylogenetic analyses

# exiting qiime2-2018.8 environment

```
source deactivate qiime2-2018.8
```
